# Dual‐view scout scans with deep learning for ultra‐low dose attenuation correction in PET

**DOI:** 10.1002/mp.70647

**Published:** 2026-08-18

**Authors:** Florence M. Muller, Margaret E. Daube‐Witherspoon, Michael J. Parma, Amy E. Perkins, Peter B. Noël, Christian Vanhove, Stefaan Vandenberghe, Joel S. Karp

**Affiliations:** ^1^ Department of Electronics and Information Systems Faculty of Engineering and Architecture Ghent University Ghent Belgium; ^2^ Department of Radiology University of Pennsylvania Philadelphia Pennsylvania USA; ^3^ Philips Healthcare Orange Village Ohio USA

**Keywords:** attenuation correction, CT‐less PET, deep learning, image generation

## Abstract

**Background:**

Accurate attenuation correction (AC) is essential for quantitative positron emission tomography (PET). Conventional CT‐based AC provides reliable attenuation (μ‐) maps but adds radiation, introduces PET/CT misalignment artifacts, and is unavailable on stand‐alone PET systems. Existing deep learning (DL) methods use non‐attenuation corrected (NAC) PET data to predict CT‐like or directly AC‐PET images, but their dependence on emission characteristics limits generalizability across tracers and anatomical coverage, especially for long axial field‐of‐view PET.

**Purpose:**

We propose incorporating tissue‐density information from dual‐view scout radiographs as supplementary input to NAC PET, acquired with a clinically significant radiation reduction relative to standard low‐dose CT.

**Methods:**

Two DL methods were evaluated for generating transmission (Tr) images as surrogate μ‐maps for PET AC: (1) (NAC)‐to‐(Tr), using coronal and sagittal NAC PET slices, and (2) (NAC + Scout)‐to‐(Tr), combining NAC PET with anterior‐posterior and lateral scout views. 53 research scans across four tracers from the PennPET Explorer were used for training and testing, with three additional tracers included for testing.

**Results:**

(NAC + Scout)‐to‐(Tr) improved quantitative accuracy, reducing NRMSE% to < 10% (versus up to 18% for (NAC)‐to‐(Tr)) and SUV biases to within ‐10%, with significant reductions in brain, liver, and muscle compared to (NAC)‐to‐(Tr). Out‐of‐distribution evaluation confirmed generalizability, maintaining SUV biases below 5%‐8% versus 10%‐13% for (NAC)‐to‐(Tr). In a longitudinal biodistribution study, the scout‐guided model showed consistent performance across repeat scans.

**Conclusions:**

By leveraging routinely acquired scout scans, this approach enables accurate quantitative PET reconstruction without a full CT, substantially reducing radiation dose and misalignment artifacts.

## 1 INTRODUCTION

In positron emission tomography (PET), accurate quantification of the radiotracer distribution critically depends on PET data corrections (attenuation, scatter, randoms, normalization). Attenuation correction (AC) in PET relies on the generation of an attenuation (μ‐) map, which is then used during image reconstruction to account for the loss of coincidence detections due to photon attenuation. The integration of computed tomography (CT) and PET has become standard clinical practice. A low‐dose 3D CT is routinely acquired to provide anatomical correlation of PET uptake and to generate μ‐maps based on tissue density.^1^ While AC itself can be performed using much lower CT dose levels,^2^ clinical protocols often employ higher doses (e.g., ∼25% of a full‐dose diagnostic CT) to improve anatomical localization. CT‐based AC presents certain limitations. First, CT scans contribute an additional radiation dose. In research settings, where PET/CT images may be acquired at multiple time‐points in a single session (or for follow‐up studies), the CT is often used solely for AC, raising concerns about unnecessary radiation exposure.^3^ Second, spatial mismatches between PET and CT, caused by respiratory motion or gross body movements between the two scans, frequently result in image artifacts and quantification biases. Finally, CT‐based AC is not available for stand‐alone PET systems, limiting its utility in such imaging scenarios, for example brain scanning.^4^

These challenges have driven interest in alternative (“CT‐less”) correction methods that eliminate the need for a separate CT scan. Before PET/CT, the gold standard for obtaining μ‐maps involved external transmission sources with a rotating Germanium‐68 rod source (coincidence‐based), which required long scans to reduce noise in the μ‐map, but became more practical as a post‐injection technique,^5^ or with a Cesium‐137 point source (singles‐based).^6^, ^7^ Other methods include emission‐based joint reconstruction of activity and attenuation maps.^8^, ^9^, ^10^, ^11^ For algorithms, such as MLAA (maximum likelihood activity and attenuation^10^), incorporating time‐of‐flight (TOF) information improves robustness to data inconsistencies.^12^ Defrise et al.^13^ established that TOF‐PET can determine the solution of joint reconstruction up to a scaling factor. However, MLAA still faces slow convergence, noisy μ‐maps, and crosstalk; these issues are exacerbated under low‐count conditions and by radiotracer‐dependent variability.^8^, ^9^, ^10^, ^11^ Another CT‐less AC method leverages the intrinsic background radiation of lutetium‐based scintillators in PET. Rothfuss et al.^14^ showed that this intrinsic Lu‐based radiation can serve as an internal transmission source, distinguishable from emission events by TOF differences and characteristic energy peaks. While promising, performance depends on having sufficient transmission counts to reliably separate from emission events.

In recent years, there has been growing interest in deep learning (DL) solutions for quantitative PET without relying on CT for AC.^15^, ^16^, ^17^, ^18^, ^19^, ^20^, ^21^ The majority of DL methods reported in the literature use non‐corrected PET data as input to either predict a CT‐like image for integration into the image reconstruction or to directly generate a fully corrected PET image. Comparative studies^22^, ^23^ suggest that indirect methods can generally achieve superior quantitative performance, albeit at the cost of additional reconstruction steps and scatter estimation (typically via single scatter simulation). Although direct prediction offers faster inference, it relies entirely on the neural network to jointly perform AC and scatter correction and is implicitly tied to the reconstruction pipeline used during training. In contrast, predicting a CT‐like image provides an interpretable intermediate output that can be inspected for artifacts and may aid anatomical localization, supporting its suitability for clinical use.^4^ Others have proposed combining DL with MLAA and/or lutetium background‐based approaches,^24^, ^25^, ^26^ aiming to improve the fidelity of the μ‐map, which is then, for example, used to initialize the transmission estimation or to serve as a global constraint to obtain more accurate attenuation values from MLAA. More recent studies^27^, ^28^ have shown that DL‐based denoising of MLAA‐derived μ‐maps, following initialization from intrinsic lutetium transmission (Lu‐Tr) data, can achieve image quality approaching CT‐AC accuracy, even at ultra‐low emission doses (<10 MBq). However, the Lu‐Tr data were acquired in a separate 5‐min scan following the PET scan using wider energy and coincidence timing windows, which may lead to emission‐transmission misalignment.

A variety of neural networks have been explored for CT‐less AC, including convolutional autoencoders, U‐Net, Generative Adversarial Networks (GANs), CycleGANs, and, more recently, diffusion models. While these approaches have shown promise, most have often focused on single tracer studies^15^, ^16^, ^17^, ^18^, ^19^ and/or region‐specific applications (e.g., brain imaging).^19^, ^20^, ^21^ A few studies^29^, ^30^, ^31^ have investigated cross‐tracer DL‐AC strategies. Toyonaga et al.^29^ studied DL‐based μ‐map prediction for multiple oncology tracers (^18^F‐FDG, ^68^Ga‐DOTATATE, and ^18^F‐Fluciclovine), including whole‐body image quality assessment and tumor quantification, though tracer‐specific networks were still trained for each tracer. Hou et al.^31^ further evaluated cross‐tracer generalizability, investigating whether models trained on one tracer (e.g., ^18^F‐FDG) could be adapted to others such as ^68^Ga‐DOTATATE and ^18^F‐Fluciclovine. They used MLAA‐derived outputs as network inputs, comparing tracer‐specific and mixed‐tracer training methods. Similarly, Sun et al.^30^ explored cross‐tracer training strategies using ^18^F‐FDG, ^18^F‐FAPI, and ^68^Ga‐FAPI to directly generate an attenuation‐ and scatter‐corrected PET image from non‐corrected PET data. Despite these advances, achieving robust cross‐tracer generalizability remains challenging. Emission‐only DL‐AC methods are inherently sensitive to variations in count statistics, tracer distributions, which may differ substantially across protocols and imaging time‐points, especially in studies with wide anatomical coverage enabled by long axial FOV (LAFOV) PET. For denoising applications, prior studies have mitigated such limitations by incorporating, for example, MRI‐derived anatomical information into DL frameworks, thereby guiding PET image enhancement and improving the signal‐to‐noise ratio compared to PET emission data alone.^32^, ^33^ This supports the adoption of analogous strategies to improve generalizability of DL‐AC performance by exploiting additional inputs that provide structural context.

In this study, we propose leveraging scout images, 2D radiographic projections acquired for scan planning using the CT X‐ray source and detector, that offer useful tissue density information at a significantly lower radiation exposure than a standard low‐dose CT used for AC. Although the extent of dose reduction varies across protocols, clinically meaningful reductions to <10% compared with low‐dose CT can be achieved when using dual‐view scouts.^34^ Prior studies in CT^35^, ^36^, ^37^, ^38^, ^39^, ^40^ have explored the use of scout images with DL to predict 3D CT image volumes (investigating ultra‐sparse sampling and CT dose reduction) or generate patient attenuation models for dose prescription/modulation. However, these approaches have primarily focused on specific anatomical regions (e.g., thorax, head). Here, we extend this concept for AC in whole‐body PET using data from a long AFOV PET scanner. We introduce a DL‐based framework that combines non‐attenuation corrected (NAC) PET with dual‐view scout images (anterior‐posterior and lateral) in a dual‐encoder network, enabling AC without the need for a full CT acquisition. In this work, we provide a comprehensive multi‐tracer evaluation, assessing performance across seven tracers and multiple whole‐body imaging protocols that encompass diverse uptake patterns and distributions.

## 2 METHOD

### 2.1 Datasets and image reconstruction

53 PET/CT datasets were retrospectively collected from four different protocols, each with a different radiotracer. These studies were acquired on the 142 cm AFOV PennPET Explorer^41^ integrated with a dual‐layer spectral CT scanner (Philips IQon, Philips Healthcare, Netherlands)^42^: 16 ^18^F‐labeled fluorodeoxyglucose (^18^F‐FDG),^43^, ^44^ 12 ^18^F‐labeled nitric oxide synthase (^18^F‐NOS),^45^ 14 ^18^F‐labeled FluorThanatrace (^18^F‐FTT),^46^, ^47^, ^48^ and 11 ^11^C‐labeled carfentanil (^11^C‐CFN)^49^ scans (Table 1). All datasets were acquired as dynamic PET scans starting at the time of injection (0 min), with total scan durations ranging from 60 to 120 min (depending on the tracer). For the present work, static emission images (10‐min duration) were reconstructed from each dynamic dataset at tracer‐specific post‐injection time‐points, as summarized in Table 1 and illustrated in Figure S‐1. Our choice of tracers and reconstruction framings was motivated to maximize variations in late‐phase tracer distributions across different anatomical regions for training and testing our DL models. Human studies were approved by the University of Pennsylvania Institutional Review Board, and study participants signed informed consent.

**TABLE 1:** Overview of the four tracers included in training and testing. Mean body mass index (BMI) and injected activities (± standard deviation) are reported. Subjects were between 18 and 80 years of age, in accordance with the approved UPenn Institutional Review Board protocol. For each tracer, extra information on the study is provided, including the post‐injection time‐point that corresponds to the start of the 10‐min static frame reconstructed from the full dynamic acquisition.

Tracer	BMI (kg/m^2^)	Dose (MBq)	Number of datasets	Imaging use on PennPET explorer
^18^F‐FDG	25.7 ± 3.8	348 ± 26	5	Datasets were obtained at 90‐100 mins p.i. from a study in subjects with epilepsy.^43^
			11	Datasets were obtained at 50‐60 mins p.i. from studies investigating glucose metabolism in alcohol use disorder.^44^
^18^F‐NOS	22.9 ± 5.0	183 ± 58	12	Datasets were obtained at 20‐30 mins p.i. from a study with users of electronic and combustible cigarettes, aimed at characterizing oxidative stress and inflammation in lungs.^45^
^18^F‐FTT	27.1 ± 3.6	375 ± 29	14	Datasets were obtained at 60‐70 mins p.i. from studies evaluating PARP (Poly‐(ADP)‐ribose‐polymerase) expression in patients with breast, ovarian, or metastatic prostate cancer.^46^, ^47^, ^48^
^11^C‐CFN	24.9 ± 3.9	159 ± 33	11	Datasets were obtained at 30‐40 mins p.i. from a study on μ‐opioid receptor imaging at baseline and after naloxone pre‐treatment.^49^

Each dataset included (i) a non‐attenuation corrected PET image (NAC PET), (ii) a CT‐derived transmission (Tr) image, (iii) a PET image reconstructed with CT‐based AC and TOF‐SSS scatter estimate,^50^ and (iv) dual‐view radiographic projections (anterior‐posterior and lateral scout), which are routinely acquired as part of clinical routine. The NAC PET images were reconstructed into 4‐mm isotropic voxels using the list‐mode TOF ordered subset expectation maximization algorithm (LM TOF OSEM, 3 iterations, 25 subsets)^51^, ^52^ with normalization correction and a delays‐based randoms correction, but without AC nor scatter correction. Tr images were transformed into 4‐mm isotropic voxels to match the NAC PET voxel dimensions. All AC PET images, whether reconstructed with the CT‐derived μ‐map or a DL‐based surrogate, were obtained by LM TOF OSEM (5 iterations, 25 subsets, 4‐mm isotropic voxels)^51^, ^52^ including all data corrections. For this study, the dual‐layer spectral CT scanner was used solely to provide conventional (non‐spectral) dual‐view scout data (network input) and CT‐rescaled Tr images (network target). Under our current protocol, each scout view exposed the patient to 0.088 mGy, and the 3D CT resulted in an average dose of about 4 mGy (patient‐weight dependent). Using dual‐view scouts corresponds thus to an approximate 22‐fold reduction in radiation dose compared to the 3D CT. While the IQon Spectral CT scanner can produce good quality images for both the scouts and 3D CT at somewhat lower doses than routinely used, this comparison illustrates the potential for significant dose reduction with scout views.

### 2.2 Preprocessing

All studies were visually inspected, and datasets showing PET/CT misalignment or motion artifacts (e.g., “banana” artifact), as well as studies with gross body motion, causing misalignment between the NAC PET (input) and Tr (target) images, were removed from the database. In total, 84 studies were collected, of which 31 were excluded based on these criteria, resulting in 53 studies included for model training and testing. From the excluded datasets, three cases were subsequently selected to illustrate the benefit of the DL‐AC methods in mitigating motion‐induced artifacts; these examples will later be shown in Figure 7. For these 53 studies, the NAC PET and Tr image volumes (both reconstructed with 4‐mm voxels) were thus aligned and defined on the same spatial grid.

Scout scans, acquired for patient position verification, are most often axially misaligned with subsequent PET/CT scans and may differ in axial coverage (i.e., mismatched fields‐of‐view). To address this, axial realignment of the scout images to the NAC PET images was performed using anatomical landmark matching. The head position was identified in both the scout image and the coronal NAC PET maximum intensity projection (MIP) image, and the resulting axial offset was applied consistently to both the anterior‐posterior and lateral scout views. The scout images were then shifted and cropped to match the axial field‐of‐view of the NAC PET. Depending on the relative offset, this involved either trimming inferior portions of the scout image or padding with zeros. No explicit image registration other than the axial realignment was applied.

Note that the acquired CT images include not only patient anatomy but also the scanner bed and additional external components such as pillow, blanket, mat, and support structures. During preprocessing, the rigid scanner bed was removed from the target CT‐rescaled Tr images using a separately acquired bed template scan. In contrast, the remaining external components are deformable and highly patient‐/scan‐specific and therefore could not explicitly be modelled. At inference, the predicted DL‐Tr images were subsequently combined with the scanner bed template prior to reconstruction, whereas for the other components this depended on the ability of the DL model to infer these structures from the available network inputs. Figure S‐2 illustrates an example of Tr images before and after scanner bed removal, demonstrating that other external components remain present in the training targets.

### 2.3 Deep learning frameworks: networks and training

**FIGURE 1:**
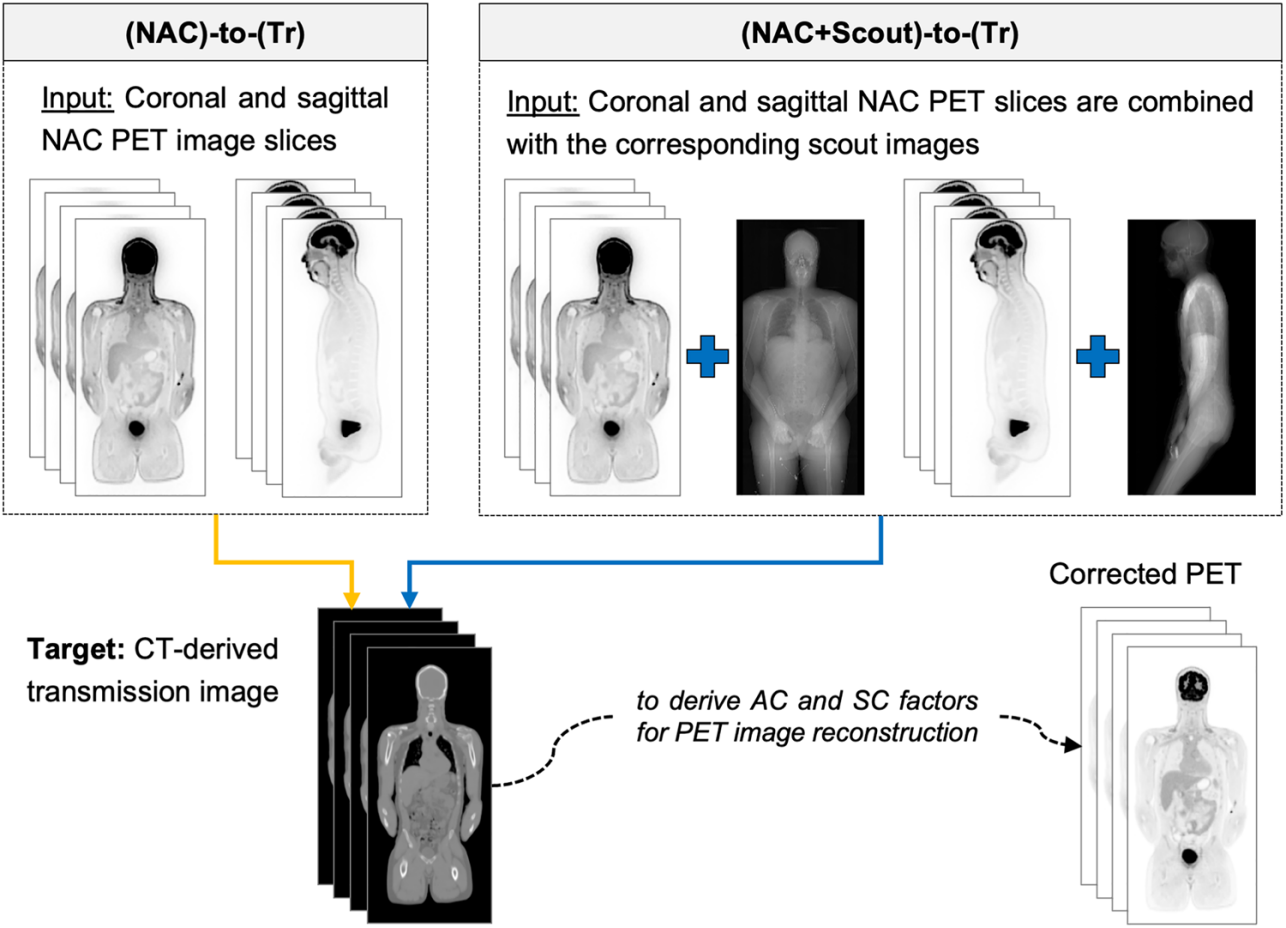
Schematic of the DL frameworks investigated in this paper for the prediction of a transmission (Tr) image used as surrogate μ‐map for attenuation correction (AC) and scatter correction (SC) in PET. Each dataset includes dual‐view scout images (shown in grey scale), a non‐AC (NAC) PET image (shown in inverse grey scale), a CT‐rescaled Tr image (target) and a PET image reconstructed with CT‐based AC (reference).

This paper compared two DL methods (Figure 1) for generating Tr images that are then utilized to derive surrogate μ‐maps for AC in PET image reconstruction. Both DL models adopt a 2D encoder‐decoder architecture and explore two network input configurations: (1) NAC‐only, using coronal and sagittal image slices, and (2) NAC + Scout, combining the NAC PET slices with the corresponding scout images. Note, a 2D framework was intentionally selected to maintain architectural consistency between the (NAC)‐to‐(Tr) and (NAC + Scout)‐to‐(Tr) approaches, since scout images are inherently acquired as 2D projections and their incorporation into a fully 3D framework would require additional projection‐to‐volume integration or interpolation strategies. Using a shared 2D architecture therefore enabled a more controlled evaluation of the contribution of scout images. In both models, the target training is the 3D CT‐rescaled Tr image.

Hyperparameter tuning was performed using the Optuna optimization framework^53^ in Python, exploring variations in network architecture (e.g., number of layers and input features, down‐ and up‐sampling operations, activation functions) and training settings (e.g., learning rate, loss function), which are further detailed in Table S‐1. A fixed training‐validation data split was defined a priori, with all training and validation datasets used throughout the Optuna optimization process, while an independent test set was reserved for final model evaluation. Instead of testing all combinations blindly, Optuna selectively searches subsets of the parameter space based on earlier trial decisions. A total of 65 Optuna trials were conducted. In this paper, we report the final best‐performing model, defined by the lowest validation loss and confirmed through qualitative image assessment, as a detailed presentation of the full optimization process is beyond the intended scope.

#### 2.3.1 (NAC)‐to‐(Tr)—network structure

**FIGURE 2:**
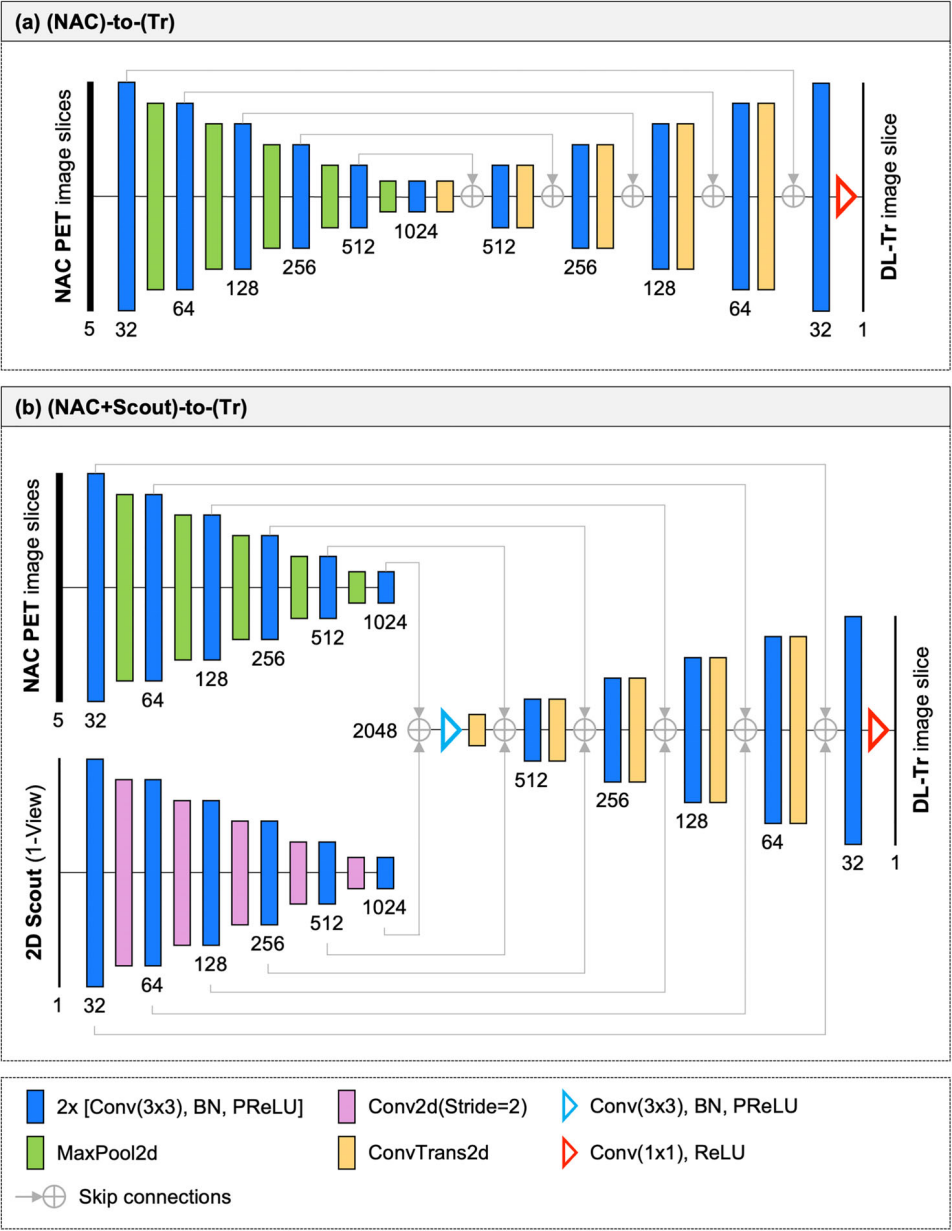
Schematic overview of the two networks: (a) (NAC)‐to‐(Tr): A U‐Net with a 2D encoder‐decoder architecture that takes as input 5 NAC PET image slices to predict the corresponding middle Tr image slice. (b) (NAC + Scout)‐to‐(Tr): A dual‐branch with separate 2D encoders for the NAC PET and scout image inputs, followed by a shared decoder.

A 2D U‐Net (Figure 2a) was used to map NAC PET image slices (coronal and sagittal orientations) to the corresponding Tr image slices. The encoder processes five consecutive slices (to capture inter‐slice information) at the input layer and expands feature maps from 32 to 1024 using 2D convolutional layers, each followed by batch normalization and parametric rectified linear unit (ReLU) activation. Down‐sampling is performed with max‐pooling, and up‐sampling with 2D transposed convolutions. Skip connections are integrated between the encoder and decoder layers. The output layer applies a 1 × 1 convolution followed by a ReLU activation to enforce positive voxel values in the prediction. The network comprises about 49.6 million trainable parameters.

#### 2.3.2 (NAC + Scout)‐to‐(Tr)—network structure

The model adopts a dual‐branch design (Figure 2b) with two separate encoders to extract modality‐specific features: one for the NAC PET slices and the other for the scout images. Each coronal and sagittal NAC PET image slice is paired with the same 2D scout acquired from the anterior‐posterior or lateral view, respectively. The encoder branch with the NAC input shares the same topology as the encoder in the (NAC)‐to‐(Tr) network. The NAC‐branch was initialized to the pretrained weights from the (NAC)‐to‐(Tr) encoder. The Scout‐branch encoder follows the same encoding depth, expanding feature maps from 32 to 1024 through 2D convolutional layers, with batch normalization and parametric ReLU. Based on the outcomes from the hyperparameter optimization process, stride convolutions are used for down‐sampling. Latent features from both encoder branches are concatenated and passed through a convolutional block that reduces the channel dimension from 2048 to 1024. The decoder employs 2D transposed convolutions with skip connections incorporated from the corresponding layers of both encoders. Also here, the output layer applies a 1 × 1 convolution followed by a ReLU activation to enforce positive voxel values in the prediction. The network has about 96.8 million parameters.

#### 2.3.3 Training settings

The 53 datasets were split at the patient level into 37 training, eight validation, and eight testing datasets. The validation and testing sets each included two datasets per tracer, resulting in the following number of training datasets per tracer: 12 ^18^F‐FDG, 8 ^18^F‐FNOS, 10 ^18^F‐FTT, and 7 ^11^C‐CFN. All slices from a given patient dataset were assigned exclusively to a single subset (training, validation, or testing), thereby preventing data leakage between subsets.

For both the (NAC)‐to‐(Tr) and (NAC + Scout)‐to‐(Tr) models, the same training configuration was used. The Adam optimizer, without learning rate scheduler, was implemented with a learning rate of 1E‐4 and batch size of 8. The initial training phase used the mean squared error (MSE) to establish a solid baseline model emphasizing voxel‐wise fidelity. The resulting MSE‐trained network was then refined via transfer learning using a composite loss of two equally weighted terms: (i) MSE, to preserve voxel‐level accuracy, and (ii) a perceptual loss computed from intermediate feature activations of a pre‐trained VGG16 network^54^ (specifically: layers 3, 8, 15, and 22 in the PyTorch implementation were used). Incorporating these multiple depths encouraged the network to match the target image not only at the voxel‐level, but also in terms of structural coherence. Early stopping was applied if the validation loss did not improve for 20 consecutive epochs.

### 2.4 Performance evaluation

#### 2.4.1 Evaluation of in‐distribution test data

From the 53 datasets, eight were left aside for testing with two test cases per tracer (^18^F‐FDG, ^18^F‐NOS, ^18^F‐FTT, ^11^C‐CFN). Performance was first evaluated on these eight in‐distribution test cases. Qualitatively, the DL‐generated Tr images were visually compared to CT‐derived Tr, and voxel‐wise SUV difference images were created between PET images reconstructed with the DL‐Tr and CT‐AC PET images. The slices shown in the difference images were selected to illustrate systematic, though not necessarily the maximum, differences. Images were further quantified using the normalized root mean squared error (NRMSE%) computed across five anatomical regions (Head & Neck, Chest, Abdomen, Pelvis, Extremity) to assess overall image quality between DL‐based and CT‐based images. These regions were defined using fixed axial boundaries, which were semi‐automatically derived from the CT‐based Tr image, rather than from atlas‐based or manual segmentations (see Figure S‐3). Volumes of interest (VOIs) were placed in brain, liver, lung, and muscle tissue regions to assess quantification errors in SUV_mean_ (i.e., relative percent bias between DL‐AC and CT‐AC PET). This was performed for all eight test studies, with results shown in boxplots. Pairwise comparisons between (NAC)‐to‐(Tr) and (NAC + Scout)‐to‐(Tr) were conducted using two‐tailed paired sample t‐tests, with a significance level of 0.01.

#### 2.4.2 Evaluation of out‐of‐distribution test data

To assess generalizability, we evaluated additional PET tracer studies not included in training, using study‐specific VOIs for regional SUV‐based evaluation.

- ^18^F‐labeled Fluoroestradiol (^18^F‐FES): Two subject datasets (BMI: 24.8 and 27.2 kg/m^2^) were obtained at about 55‐70 mins p.i. from ^18^F‐FES PET protocols (167 and 223 MBq, respectively), where the ^18^F‐FES serves as a biomarker for identifying estrogen receptor‐positive metastatic breast cancer patients who are likely or unlikely to respond to endocrine therapy.^55^ Relative percent biases in SUV_max_ quantification of a breast lesion were reported.
- ^89^Zr‐labeled anti‐CD8 minibody (^89^Zr‐CD8 PET): Two research subjects (BMI: 26.6 and 27.8 kg/m^2^) were scanned for 30 mins at 24 h p.i. of an ^89^Zr‐labeled tracer (41 and 38 MBq, respectively) targeting CD8^+^ T‐cells to assess T‐cell trafficking and tumor infiltration.^56^ Relative percent biases in SUV_mean_ and SUV_max_ of the bone marrow were calculated using a VOI in the pelvis.

#### 2.4.3 Evaluation of a patient case with repeat scans for a biodistribution study

We present a case involving multiple PET/CT scans of a single subject (BMI: 23.3 kg/m^2^) as part of a biodistribution study using 252 MBq of ^18^F‐JSS‐183A, a 4‐repeat (4R) isoform tau tracer used in neurodegenerative tauopathies.^57^ Five PET scans were acquired on the same day: an initial 80‐min dynamic scan, followed by a 30‐min dynamic scan after a 20‐min break, and three additional 10‐min acquisitions at 3, 4, and 6 h p.i. Each PET acquisition was preceded by a dual‐view scout and a CT scan. For the scope of this paper, we selected 10‐min frames at 1, 2, 3, 4, and 6 h p.i. from the five PET acquisitions. For evaluation across the five datasets, the NRMSE% was calculated for four anatomical regions (Head & Neck, Chest, Abdomen, Lower Body) between DL‐based and CT‐based images. As with the in‐distribution test data, these regions were defined using fixed axial boundaries (see Figure S‐3). Pairwise comparisons between (NAC)‐to‐(Tr) and (NAC + Scout)‐to‐(Tr) were conducted using two‐tailed paired sample t‐tests, with a significance level of 0.01.

## 3 RESULTS

### 3.1 Evaluation of in‐distribution test data

**FIGURE 3:**
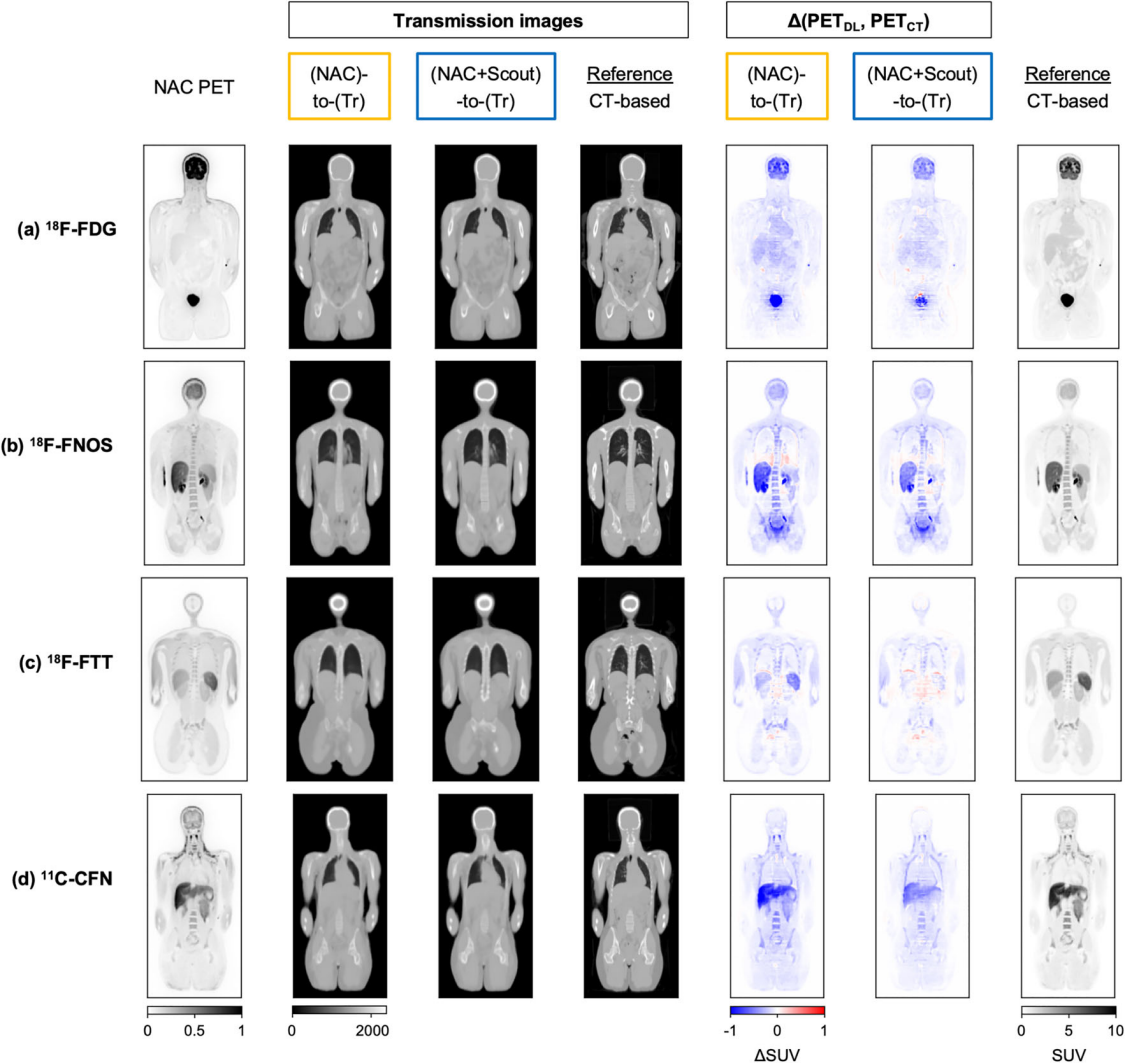
Qualitative evaluation, comparing the DL‐Tr images generated from NAC PET input only (yellow) and from NAC PET combined with dual‐view scout images (blue) against the CT‐derived Tr (reference). PET images reconstructed with DL‐Tr images are compared to the CT‐AC PET, with corresponding difference images shown as voxel‐wise SUV differences. Results are shown for the four tracers included in training (“in‐distribution”): (a) ^18^F‐FDG, (b) ^18^F‐FNOS, (c) ^18^F‐FTT, (d) ^11^C‐CFN.

**FIGURE 4:**
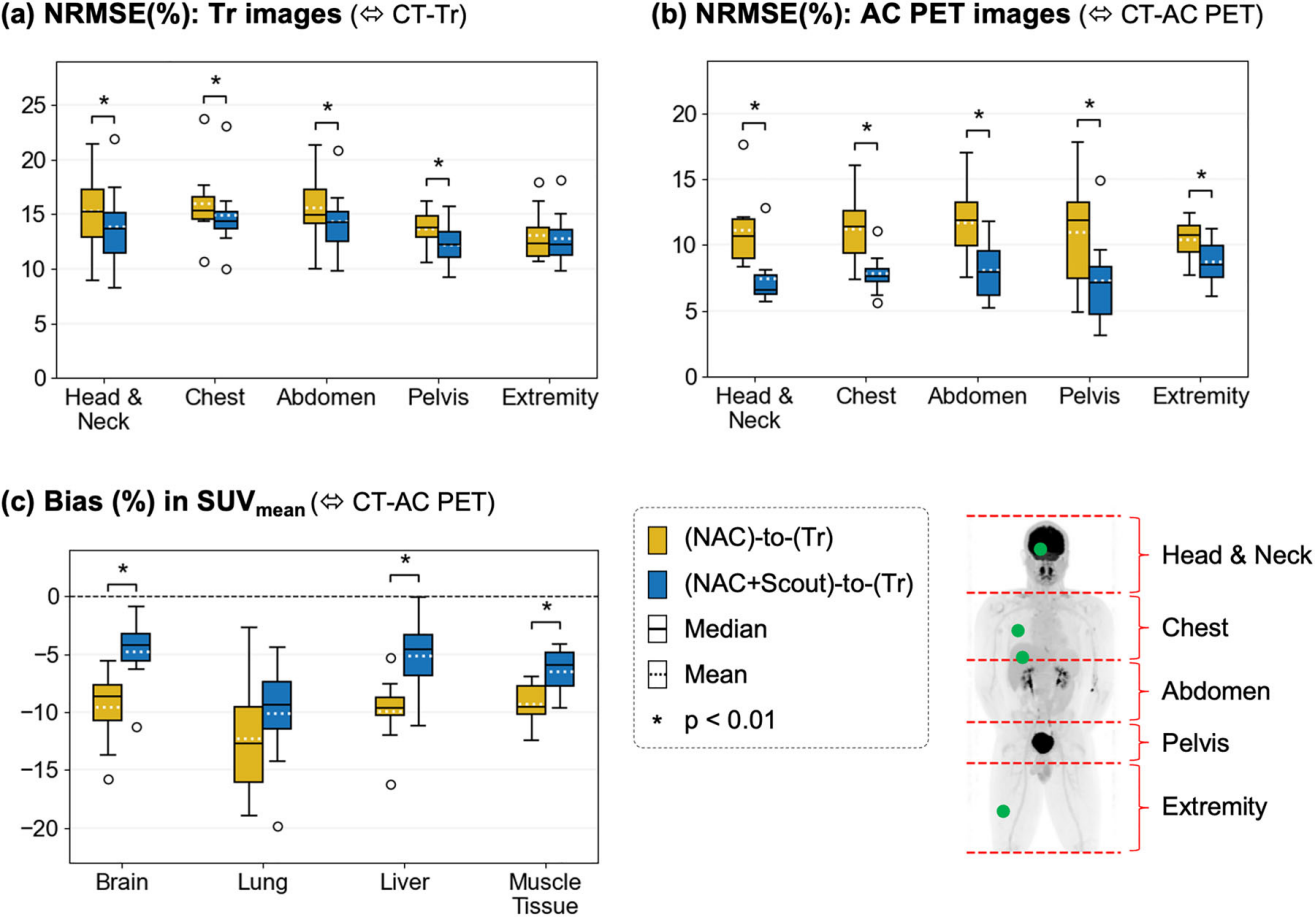
Data presented in the box plots are calculated from the eight test subjects: two per tracer study (^18^F‐FDG, ^18^F‐FNOS, ^18^F‐FTT, ^11^C‐CFN). (a) Normalized root mean squared error (NRMSE%) between DL‐Tr and CT‐Tr images, and (b) NRMSE% between the resultant AC PET images. (a‐b) Results of NRMSE% are stratified across five anatomical regions: Head & Neck, Chest, Abdomen, Pelvis, and Extremity. (c) Relative percent bias in SUV_mean_ between DL‐ and CT‐based AC PET measured in four tissue regions: Brain, Lung, Liver, Muscle (green circles). Box plots show median (black line), mean (white line), variability (whiskers), and outliers (circles). Statistical significance (*p* < 0.01) is shown for pair‐wise comparisons of (NAC)‐to‐(Tr) and (NAC + Scout)‐to‐(Tr).

Figure 3 compares Tr and PET images from DL‐ and CT‐based methods for one representative test subject from each in‐distribution tracer. Difference images are shown as ΔSUV maps to highlight systematic differences. DL‐Tr images capture body contours and major organ boundaries, but are less sharp than CT‐derived Tr, with reduced contrast and loss of fine anatomical details (e.g., rib bones, lung branches). Comparing (NAC)‐to‐(Tr) with (NAC + Scout)‐to‐(Tr) reveals that adding the scout improves delineation of bone and lung; however, predicted bone intensity remains lower than in CT‐Tr. In Figure 4a, the (NAC + Scout)‐to‐(Tr) yields significantly lower NRMSE% in head & neck, chest, abdomen, and pelvis (*p* < 0.01), with reduced variability, whereas extremities show smaller gains. Variability is characterized by box height (interquartile range) and whisker length (non‐outlier values). Difference images between PET_DL‐AC_ and PET_CT‐AC_ indicate biases within the order of ±1 SUV (<15%), with larger discrepancies for (NAC)‐to‐(Tr) than (NAC + Scout)‐to‐(Tr). Small horizontal lines in the difference maps (<1% of the mean SUV) originate from small differences in the scatter tail fitting when using CT‐based versus DL‐predicted Tr images. Both DL methods show a systematic negative bias versus CT‐AC, indicating under‐correction for attenuation. The scout‐guided model reduces this bias, as confirmed by Figure 4b, where NRMSE% for (NAC + Scout)‐to‐(Tr) is significantly lower in all regions (*p* < 0.01) with less variability, reflected by shorter boxes and whiskers.

SUV‐based quantification (Figure 4c) further shows that the addition of the scout input reduces relative SUV_mean_ bias across all tissues, keeping biases within −10% except in the lung. For brain, liver, and muscle VOIs, bias reduction with (NAC + Scout)‐to‐(Tr) is significant (*p* < 0.01), compared to (NAC)‐to‐(Tr). The lung shows the largest variability, with broader interquartile ranges and no significant difference between the two DL models. Consistent with qualitative results, the bias direction across all tissue regions is negative, indicating a systematic under‐estimation relative to CT‐AC.

### 3.2 Evaluation of out‐of‐distribution test data

**FIGURE 5:**
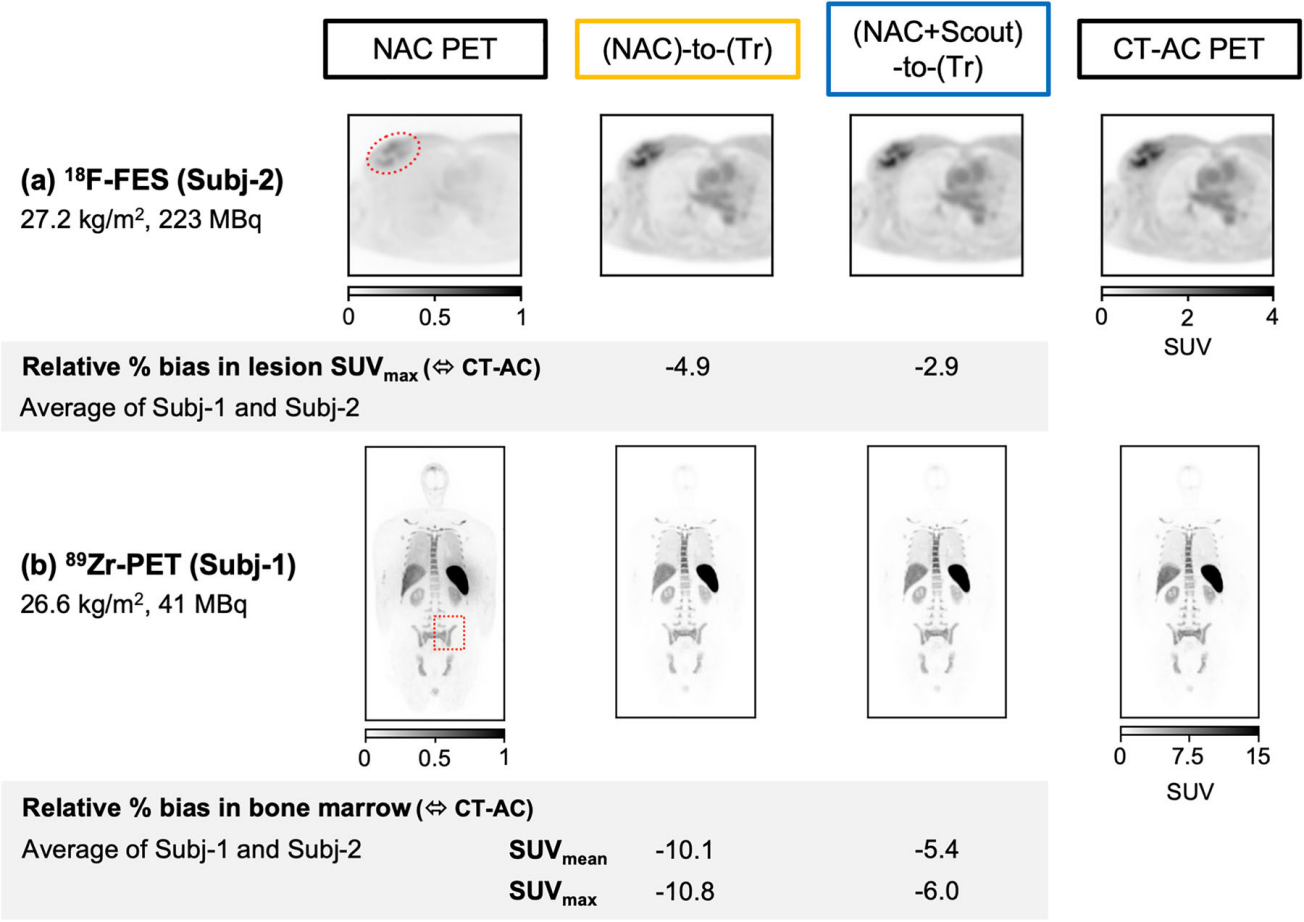
Evaluation of (NAC)‐to‐(Tr) and (NAC + Scout)‐to‐(Tr) on out‐of‐distribution testing data. (a) ^18^F‐FES PET: Comparison of AC PET images reconstructed with DL‐Tr images versus CT‐AC PET, with percent bias in lesion SUV_max_ reported as the average of the two patients. (b) ^89^Zr‐PET: Comparison of AC PET images reconstructed with DL‐Tr images versus CT‐AC PET, with percent biases in bone marrow SUV_mean_ and SUV_max_ reported as the average of the two patients.

For one of the two ^18^F‐FES PET studies Figure 5a, a representative transverse slice shows a spiculated breast lesion. The lesion morphology is maintained for the (NAC)‐to‐(Tr), (NAC + Scout)‐to‐(Tr), and CT‐AC PET images. Quantitatively, the average lesion SUV_max_ bias across both studies is below 5% in both DL‐based cases relative to the CT‐AC PET. Figure 5b shows that the ^89^Zr‐PET images reconstructed with DL‐Tr exhibit bone and soft tissue uptake comparable to CT‐AC PET, albeit with a slightly lower global intensity, most evident in the liver and pelvis. Quantitatively, the average bias in bone marrow SUV_mean_ and SUV_max_ slightly exceeds ‐10% for (NAC)‐to‐(Tr) but is reduced to ‐6% with (NAC + Scout)‐to‐(Tr) relative to CT‐AC PET.

### 3.3 Evaluation of a patient case with repeat scans for a biodistribution study

**FIGURE 6:**
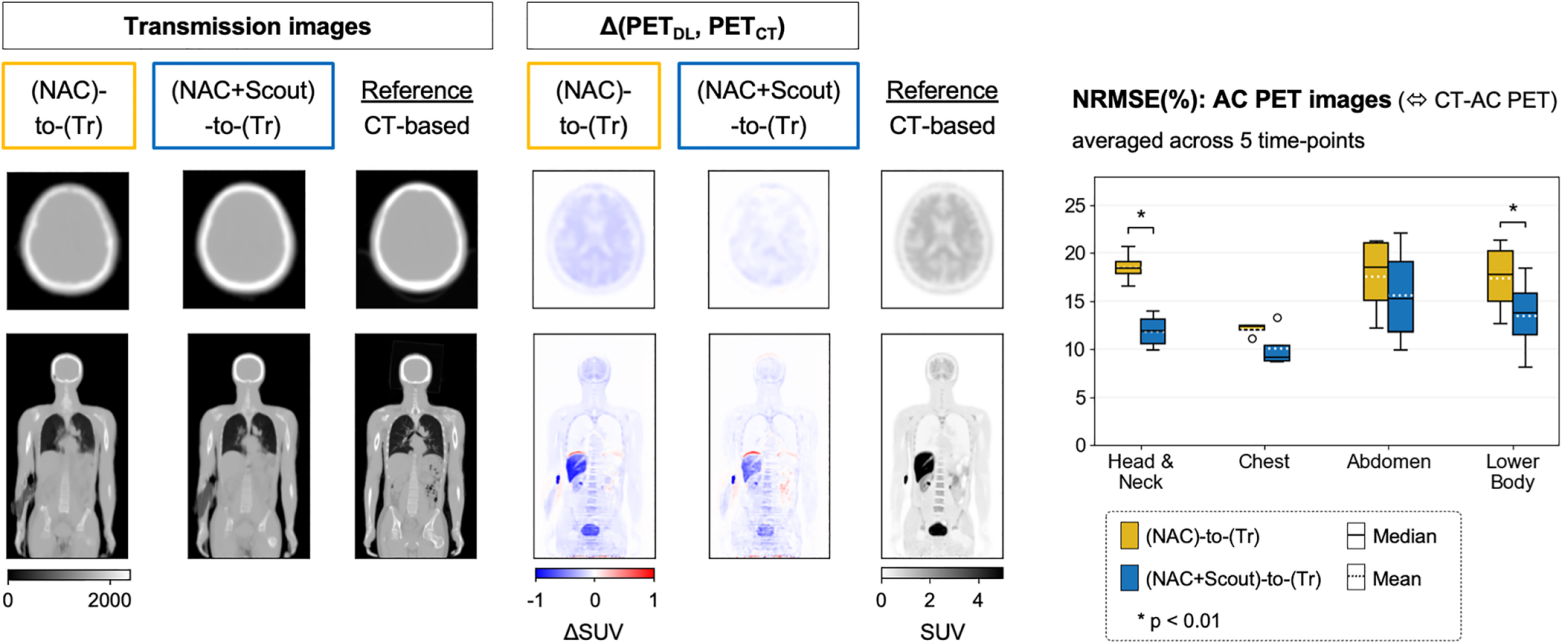
Biodistribution study with ^18^F‐JSS. Qualitative results are shown for the 1‐h p.i. scan, comparing the DL‐Tr images to CT‐Tr, as well as the reconstructed AC PET images, with corresponding difference images shown as voxel‐wise SUV differences. Results for the NRMSE% of AC PET images reconstructed with DL‐Tr relative to CT‐AC PET are reported as average across five time‐points (1, 2, 3, 4, and 6 h p.i.). Box plots show median (black line), mean (white line), variability (whiskers), and outliers (circles). Statistical significance (*p* < 0.01) is shown for pair‐wise comparisons of (NAC)‐to‐(Tr) and (NAC + Scout)‐to‐(Tr).

Figure 6 shows the results from the biodistribution study with five same‐day scans (10‐min frames at 1, 2, 3, 4, and 6 h p.i.). Tr image comparisons (Figure 6) reflect the trends seen with the “in‐distribution” tracers in Figure 4: overall morphology is preserved, but bone contrast is reduced, and lungs appear more opaque for (NAC)‐to‐(Tr) than for (NAC + Scout)‐to‐(Tr). In all the DL‐Tr images, an artifact in the right forearm is attributed to tracer trapped in the IV line, resulting in abnormally high activity in the NAC image. Difference images confirm a small overall negative bias (ΔSUV < 1) observed in previous results, along with a positive bias at the diaphragm suggesting a motion‐induced “banana” artifact. Regional NRMSE%, averaged over all five time‐points to capture variability of DL‐AC predictions across repeat scans, shows that (NAC + Scout)‐to‐(Tr) consistently reduces NRMSE%, with significant improvement in head & neck and lower body regions, and smaller, non‐significant reductions in chest and abdomen.

### 3.4 Evaluation of patient cases with motion‐induced artifacts and implants

**FIGURE 7:**
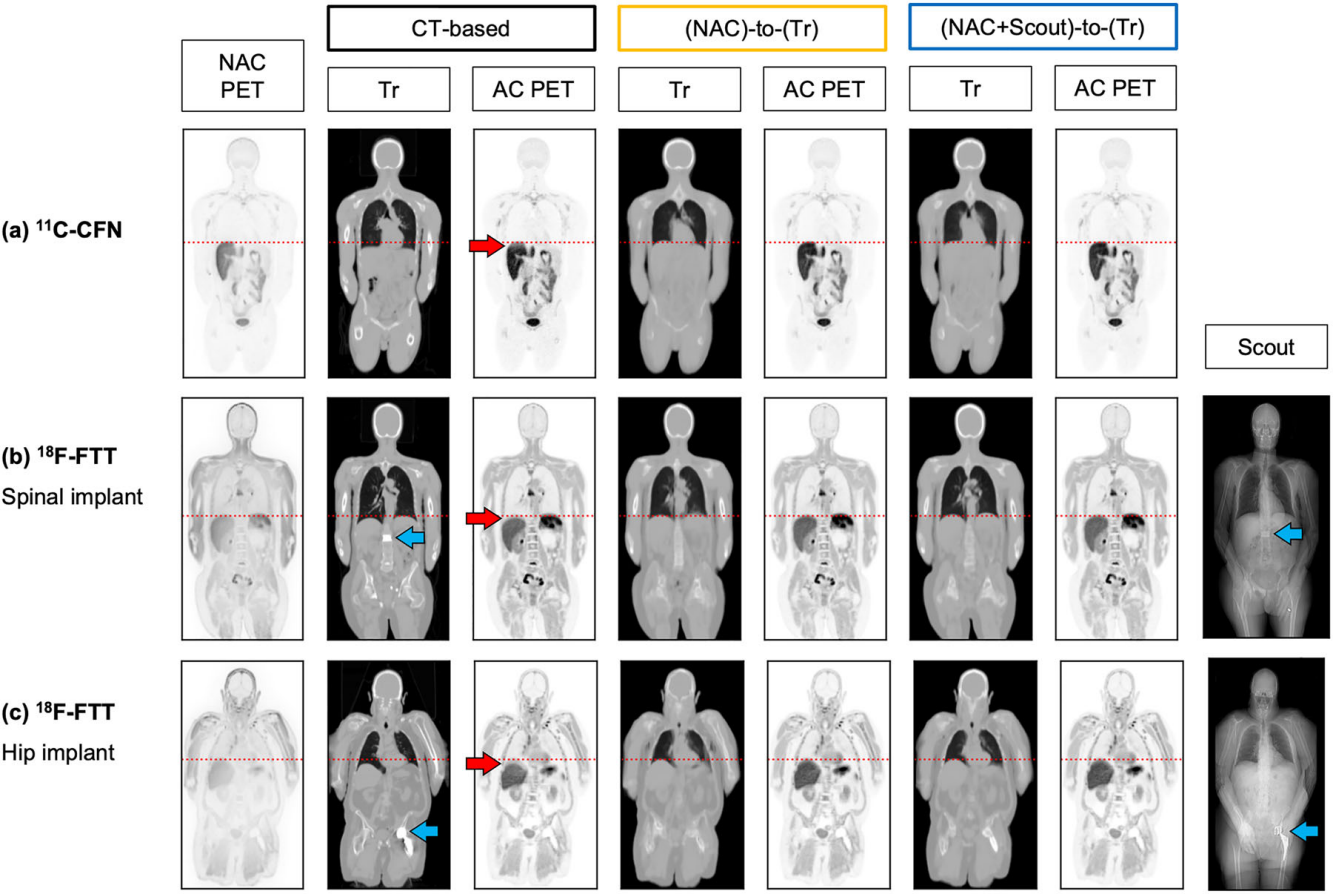
Representative patient cases where respiratory motion is identified, leading to a mismatch between the acquired CT and reconstructed PET frame, resulting in a “banana” artifact (red arrow) in the AC PET. Both DL‐AC methods generate a Tr image that is aligned to the NAC PET (red dlines), eliminating the artifact in the AC PET. (a) A ^11^C‐CFN study (35‐40 mins p.i., 185 MBq, BMI: 19.2 kg/m^2^). (b) A ^18^F‐FTT study (60‐70 mins p.i., 368 MBq, BMI: 23.1 kg/m^2^) with a spinal fusion cage (blue arrow). (c) Another ^18^F‐FTT study (60‐70 mins p.i., 430 MBq, BMI: 36.1 kg/m^2^) with a hip implant (blue arrow).

Figure 7 presents three patient test cases in which respiratory motion is evident, leading to a mismatch between the acquired CT and the reconstructed PET frame and resulting in a characteristic “banana” artifact (red arrow) in the AC PET image. Both DL‐AC methods generate Tr images that are well aligned with the NAC PET (red dotted lines), eliminating the artifact in the corresponding AC PET. The subjects shown in Figure 7b and c contain a spinal fusion cage and hip implant (blue arrow), respectively. The (NAC)‐to‐(Tr) failed to reproduce the implants visible in the CT‐derived Tr image. In contrast, the (NAC + Scout)‐to‐(Tr) partially recovered the metal hip prosthesis, clearly visible in the scout image, but did not recover the less dense (ceramic) spinal fusion cage.

## 4 DISCUSSION

In this study, two DL frameworks were evaluated for generating Tr images for PET AC, directly from NAC PET data, with and without supplementary dual‐view scout inputs. Prior CT‐less DL‐AC methods^15^, ^16^, ^17^, ^18^, ^19^, ^20^, ^21^, ^22^, ^23^ have been constrained by dependence on tracer‐specific uptake patterns, count statistics, and anatomical regions. The proposed dual‐encoder network incorporates scout images as auxiliary input, providing complementary tissue density information to enhance μ‐map estimation and reduce reliance on the emission distribution. Models were trained on four tracers and evaluated on seven tracers, without fine‐tuning, to test whether the inclusion of scout data improves robustness, analogous to prior approaches that integrate anatomical priors for PET image enhancement.^32^, ^33^

### 4.1 In‐distribution tracer evaluation

The proposed (NAC + Scout)‐to‐(Tr) framework generated anatomically consistent Tr images and enabled more quantitatively accurate PET reconstructions than the (NAC)‐to‐(Tr) framework across multiple tracers and acquisition conditions. Although DL‐Tr images were less detailed than CT‐Tr, scout integration improved depiction of bone and lung regions. Quantitative analyses confirmed that the (NAC + Scout)‐to‐(Tr) network achieved significantly lower NRMSE% and SUV biases across most regions compared to the (NAC)‐to‐(Tr) network, with reduced inter‐subject variability, indicating more generalizable performance. A systematic negative bias suggests global under‐correction for attenuation relative to CT‐AC PET. One contributing factor is the unmodeled attenuation from external components such as the pillow, blanket, or support structures. As described in Section 2.2, the rigid scanner bed was explicitly added back into the image using a separately acquired bed template scan. However, other external components are deformable and highly patient‐specific, so their attenuation contribution depends on the ability of the DL model to infer these structures from NAC PET and scout inputs. This inference task remains challenging given that attenuation prediction from non‐positron‐emitting regions is difficult to infer from emission‐only inputs. The scout‐guided framework helps mitigate this systematic bias relative to (NAC)‐to‐(Tr) by providing additional structural information, but underestimation can still occur due to incomplete recovery of these external components.

From a clinical perspective, the SUV biases observed with the (NAC + Scout)‐to‐(Tr) method (generally within 5‐8%) are below the response thresholds defined by the PERCIST (PET Response Criteria in Solid Tumors) criteria used for therapy assessment, where approximately 20‐30% changes in SUV are required for response classification.^58^ Our findings suggest that the reported biases for (NAC + Scout)‐to‐(Tr) network are unlikely to substantially affect clinical response assessment, while potentially supporting improved precision in tracking smaller longitudinal changes.

### 4.2 Cross‐tracer (out‐of‐distribution) evaluation

Out‐of‐distribution evaluation included three representative scenarios: (i) ^18^F‐FES PET, to assess lesion quantification, (ii) ^89^Zr‐CD8^+^ PET, characterized by a very different uptake pattern and low‐count conditions, and (iii) a ^18^F‐JSS PET biodistribution study with five repeat scans acquired from 1 to 6 h p.i. This enabled comprehensive assessment under diverse imaging conditions. While these in‐ and out‐of‐distribution evaluations provide initial evidence of robustness across tracers, with SUV biases below 5‐8% for (NAC + Scout)‐to‐(Tr) versus 10%‐13% for (NAC)‐to‐(Tr), the relatively small test cohort (eight in‐distribution cases and two cases per tracer for out‐of‐distribution evaluation) limits statistical power. Larger‐scale studies will be required to confirm these findings and to evaluate generalizability of the proposed approach across different scanner types and scout acquisition protocols. Prior work by Hou et al.^31^ and Sun et al.^30^ investigated cross‐tracer generalizability using single‐tracer or mixed‐tracer training with transfer learning from ^18^F‐FDG to other tracers. In contrast, the present study focused on cross‐tracer robustness achieved through an auxiliary scout‐guided network input, without transfer learning. For ^18^F‐FES, both models achieved lesion quantification within 5% of reference SUV_max_, a difference unlikely to impact clinical interpretation at higher SUV_max_ levels (Subj‐1: 8.2, Subj‐2: 10.8), but potentially more relevant near threshold SUV_max_ values (1.5‐1.8) predictive of endocrine therapy response.^59^ The ^89^Zr‐CD8^+^ study also exemplifies low‐dose delayed imaging conditions, where NAC‐only DL methods are known to be sensitive to reduced counts and increased noise. The scout‐guided framework showed improved robustness to count variability, though further systematic evaluations across a wider range of count levels are warranted.

The ^18^F‐JSS biodistribution study confirmed quantitative reliability (in terms of reduced NRMSE%) of the (NAC + Scout)‐to‐(Tr) network across repeat scans, despite variations in tracer uptake due to decay and redistribution over time. Such CT‐less DL‐AC approaches are particularly advantageous in longitudinal imaging, where repeated CT scans would otherwise increase cumulative radiation exposure. As noted in,^3^ approximately 75% of CTs in PET studies are performed primarily for AC and anatomical localization rather than diagnosis. DL‐generated Tr images, while not of diagnostic quality, provide sufficient accuracy to minimize bias during PET image reconstructions.

### 4.3 Advantages and limitations

The (NAC + Scout)‐to‐(Tr) framework offers practical advantages. Scout scans are routinely acquired for patient positioning to define the axial scan range for any CT acquisition (including ultra‐low dose scans) and contribute minimal radiation.^34^ Accordingly, the primary goal of a scout‐guided DL‐AC approach is to reduce X‐ray dose while preserving quantitative PET accuracy, without requiring a volumetric CT acquisition. This becomes particularly relevant for repeat or longitudinal studies, where radiation safety considerations limit cumulative CT exposure. In this setting, scout‐guided methods can provide clinically meaningful dose savings (<0.2 mGy instead of 4 mGy based on our current protocol for the datasets of this study), especially on older systems that lack advanced low‐dose CT capabilities. Related work in CT has reconstructed 3D image volumes from one or two 2D projections with DL for specific anatomical regions (e.g., thorax, head).^35^, ^36^, ^39^, ^40^ The (NAC + Scout)‐to‐(Tr) network extends this concept to whole‐body imaging by combining dual‐view scouts with NAC PET images to estimate 3D attenuation maps. On‐going work investigates a (Scout)‐to‐(Tr) model that predicts a μ‐map solely from scout views,^60^ thereby eliminating dependence on tracer distribution and counts and bypassing NAC reconstruction; however, a scout‐only approach does not provide inherent spatial alignment with the PET emission image and may therefore require an additional image registration step. By incorporating the NAC PET into the network input, the DL‐predicted attenuation image is aligned with the PET data, which is a practical advantage of the dual‐encoder framework.

It should be acknowledged that the (NAC)‐to‐(Tr) method could potentially benefit from a dedicated 3D architecture and may achieve improved performance. In this study, we used comparable 2D network architectures for both the (NAC)‐to‐(Tr) and (NAC + Scout)‐to‐(Tr) networks, with 5 adjacent NAC slices provided at the input layer to incorporate limited inter‐slice contextual information. The 2D framework was intentionally selected to maintain consistency with the scout input (inherently 2D images) and enable a controlled comparison between the two approaches, with the main objective to evaluate whether the inclusion of scout information improves DL‐based AC performance.

More broadly, a key motivation for CT‐free AC is to eliminate the CT entirely, which is especially relevant for dedicated PET systems without integrated CT. Although the present method relies on CT scout views and thus assumes the availability of CT hardware, the results suggest that a simple, lower‐cost source‐detector configuration could be integrated into stand‐alone PET systems to offer scout‐like (2D projection) imaging. Beyond dose reduction, emission‐derived AC methods benefit from intrinsic co‐registration between emission and Tr images, eliminating PET‐CT misalignment artifacts such as the lung‐liver “banana” artifact shown in Figure 7.

Furthermore, implants pose a particular challenge for emission‐only AC, as they lack tracer uptake, yet cause strong attenuation. In Figure 7b and c, the (NAC)‐to‐(Tr) network failed to reproduce the implants visible in CT‐Tr, while the (NAC + Scout)‐to‐(Tr) network partially recovered the metal hip prosthesis (clearly visible in the scout image), but not the less dense (ceramic) spinal cage. This highlights the value of scout‐derived tissue density information for improving attenuation estimation in implant regions, while underscoring the need for further model refinement with broader and more explicit material density discrimination. With the availability of spectral CT on the PennPET Explorer, future work will investigate combining multiple spectral scout maps via projection‐based material decomposition to further enhance tissue differentiation and DL‐AC performance. Spectral CT enables material‐specific mapping based on energy‐dependent attenuations without extra dose,^61^ while spectral scouts can provide 2D material‐specific information and added diagnostic value compared to conventional scouts (e.g., bone mineral density assessment^62^, ^63^, ^64^).

## 5 CONCLUSIONS

We compared two DL methods for generating transmission (Tr) images as surrogate μ‐maps for PET reconstruction. Our results showed that a neural network combining non‐attenuation corrected emission data (NAC PET) with dual‐view scout images (2D radiographic projections providing tissue density information) significantly outperformed a network using NAC PET alone. The (NAC + Scout)‐to‐(Tr) network achieved more robust performance in terms of lower NRMSE% across multiple tracers and anatomical regions. SUV bias and inter‐subject variability in the brain, liver and muscle tissues were significantly reduced. A clinical advantage of the scout‐guided method is that it enables operation of a PET/CT system using only scout scans, thereby reducing overall radiation exposure. Limitations remain in accurately representing bone, lung, and implant structures. Future work will explore the potential of spectral scout scans with material‐specific mapping to improve tissue discrimination. Beyond dose reduction, DL‐AC may also mitigate motion‐induced PET/CT misregistration, making the (NAC + Scout)‐to‐(Tr) DL approach a practical, low‐dose alternative to CT‐based AC for quantitative whole‐body PET, particularly valuable in longitudinal studies.

## CONFLICT OF INTEREST STATEMENT

Amy E. Perkins is an employee of Philips Healthcare. The other authors have no relevant conflicts of interest to disclose.

## Supporting information



Supporting Information
