# Supplementary material for: Dual‐view scout scans with deep learning for ultra‐low dose attenuation correction in PET
Source: Med Phys. 2026 Aug 18;53(9):e70647. doi: 10.1002/mp.70647 (PMC13486921; doi:10.1002/mp.70647)
Supplement: Supplementary file 1 [file MP-53-0-s001.doc]

**Supplementary Material for: “Dual-View Scout Scans with Deep Learning for Ultra-Low Dose Attenuation Correction in PET”**

**Methods - Datasets**

**Figure S-1:** Representative dynamic PET image slices are shown at multiple time-points post-injection (p.i.) for **(a)** ^18^F-FDG, **(b)** ^18^F-FTT, **(c)** ^18^F-NOS, **(d)** ^11^C-CFN. Images are displayed on the same SUV scale to note differences in relative organ uptake. For each tracer, a single late-phase time-point p.i. (10-min duration, outlined in red) was selected from the dynamic dataset for static image reconstruction, and used for training and evaluation.

**Methods – Scanner Bed Removal for CT-based Tr image during Preprocessing**

**Figure S-2:** Example of CT-based Tr images before and after scanner bed removal during preprocessing. **Left:** Tr images including the rigid scanner bed and additional external components. **Right:** Corresponding Tr images after removal of the scanner bed using a separately acquired bed template scan. While the scanner bed was explicitly removed, other external components such as the blanket, pillow, mat, and support structures remained present.

**Methods - Optuna Optimization Process**

**Table S-1:** Hyperparameter search space used for Optuna Optimization

| **Hyperparameter** | **Type** | **Search Range / Candidates** |
| --- | --- | --- |
| Number of input slices | Integer | 1-9 (step = 2) |
| Number of initial feature channels at input layer | Integer | [8, 16, 32, 64] |
| Network depth, i.e., number of layers | Integer | [3, 4, 5] |
| Down-sampling strategy | Categorical | [MaxPool, StridedConv] |
| Up-sampling strategy | Categorical | [Bilinear Upsample, ConvTranspose] |
| Activation function | Categorical | [ReLU, LeakyReLU, PReLU, Tanh, ELU, GELU] |
| Optimizer learning rate | Continuous | [1x10^-5^, 1x10^-3^] (log-uniform) |
| Learning-rate decay factor | Continuous | [0.85, 1.0] |

To evaluate the hyperparameter optimization process, several Optuna visualization tools were used:

- **Parameter importance plots** were employed to quantify the contribution of each hyperparameter to variability in the objective (loss) function, enabling assessment of model sensitivity and prioritization of influential parameters while fixing less important ones.
- **Slice plots** were used to analyze the effects of individual hyperparameters on the objective (loss) value while marginalizing over others.
- **Parallel coordinate plots** were used to visualize relationships across trials, facilitating identification of parameter combinations associated with improved performance, correlations between parameters, and suboptimal regions of the search space.
- **Rank plots** were used to show the distribution of hyperparameter values across trials ranked by objective value, providing a complementary view of how parameter choices relate to optimization performance.

**Methods - Definition of Anatomical Regions for NRMSE% Analysis**

**Figure S-3:** The normalized root mean squared error (NRMSE%) was computed within anatomical regions defined from the whole-body image volume. Regions were semi-automatically determined using axial slice boundaries derived from the CT-based Tr image. Thresholding operations were applied to identify key anatomical transition points, such as the head, shoulder, and lung-liver boundaries. Pelvis and extremity regions were defined using fixed axial offsets relative to the abdomen start slice. Subject-specific manual adjustments were subsequently performed, when automated boundary detection was inaccurate.
